# The Effect of Rotary-Die Equal-Channel Angular Pressing Process on the Microstructure, the Mechanical and Friction Properties of GW103 Alloy

**DOI:** 10.3390/ma15249005

**Published:** 2022-12-16

**Authors:** Cai Chen, Dongsheng Han, Mingchuan Wang, Ting Cai, Ningning Liang, Benoit Beausir, Huan Liu, Sen Yang

**Affiliations:** 1 Sino-French Engineer School, Nanjing University of Science and Technology, Xiaolingwei 200, Nanjing 210094, China; 2 School of Materials Science and Engineering, Nanjing University of Science and Technology, Xiaolingwei 200, Nanjing 210094, China; 3 Laboratoire d’Etude des Microstructures et de Mécanique des Matériaux (LEM3), UMR 7239, CNRS, Université de Lorraine, 57000 Metz, France; 4 College of Mechanics and Materials, Hohai University, Nanjing 210098, China

**Keywords:** RD-ECAP, Mg-Gd-Y, dynamic recrystallization, strength and ductility, friction behavior

## Abstract

In this study, the effect of rotary-die equal-channel angular pressing (RD-ECAP) on the microstructure and texture evolution of GW103 alloy is studied. RD-ECAP processes were carried out for 1, 4 and 12 passes at 450 °C. The mechanical properties and friction behavior of RD-ECAP-processed Mg-10Gd-3Y (wt%) alloy (GW103) are discussed. The results reveal that the size of dynamic recrystallized grains and second-phase particles are significantly refined to about 1.3 μm and 1 μm, respectively. The texture evolution of the processed samples is studied by X-ray diffraction and electron backscattered diffraction techniques. The multiple texture components formed are not observed after the conventional ECAP process. Moreover, different dynamic recrystallization (DRX) mechanisms are systemically analyzed and discussed in view of the texture evolution of ECAP processed samples. The final textures obtained after 12 passes are identified as two types: The C-texture type induced by continuous and discontinuous DRX, and the random texture components induced by reorientation of the initial <10$\bar{1}$0> fiber. Based on the grain refinement, precipitate strengthening and texture weakening mechanisms, a high-performance ternary alloy of Mg-Gd-Y was firstly obtained through 12 passes RD-ECAP processing, with a combination of high yield strength of 312 MPa and a high ductility of 22%. In addition, the friction behaviors are also studied. The multi-pass-processed samples exhibit a relatively lower friction coefficient under a load of 10 N at room temperature.

## 1. Introduction

As the lightest structural metals, magnesium (Mg) alloys have wide applications in the aerospace, automotive, and biomedicine industries due to their low density, high specific strength, damping capacity and good biocompatibility [1,2,3]. However, traditional Mg alloys exhibit relatively poor mechanical properties and formability at room temperature, which limits their further promotion and application in the industry. In recent decades, Mg-rare earth (RE) alloys have attracted attention due to their excellent mechanical properties by alloying with RE elements such as La, Gd, Y, Nd and Er, etc., [4,5]. Particularly, by adding both Gd and Y, the strength and ductility of Mg alloy could be improved simultaneously [6,7]. This presents the synergistic effect of excellent solid solution strengthening and age-hardening capabilities in ternary Mg-Gd-Y alloys [6,8].

As many studies have reported, the strength of Mg-Gd-Y ternary series alloys can be enhanced via casting, conventional deformation techniques and subsequent heat treatment [6,8,9,10]. Wei et al. [11] prepared a Mg-6.2Gd-3.19Y-0.39 (wt%) alloy by casting and subsequent heat treatment. The subsequent results revealed that a significant improvement in the ultimate tensile strength could be achieved by peak aging treatment. However, the ductility was dramatically reduced from 9.9% to 1.5%. Similar to the wrought Mg-Gd-Y alloys processed by conventional deformation techniques, such as extrusion and rolling, excellent strength can be obtained while the ductility still has difficulty exceeding 15% [6]. Strong basal texture is normally observed in samples processed by conventional extrusion or rolling [12]. The above problems were solved by the development of the severe plastic deformation (SPD) technique, which is considered an effective method to simultaneously improve the strength and ductility. The grain refinement process for bulk materials or powder consolidation was induced by applying the accumulated high shear strain in the SPD process [13,14,15]. Equal channel angular pressing (ECAP) and high-pressure torsion (HPT) are two typical methods that have been widely used in the research on bulk Mg alloys due to their excellent refining ability in both grains and precipitates [16,17,18,19,20]. The ECAP process is considered to have more potential industry applications compared to HPT since large-scale samples can be processed with ECAP. Continuous multi-pass deformation is difficult to achieve using the traditional ECAP mold design, which is considered a key point for the application of ECAP to larger scale industry products. The continuous process of ECAP was invented and developed [21], such as ECAP–Conform [22]; the application of this continuous ECAP process is still challenging for high-shear–strain imposing, and this technique had rare application on Mg alloy. More recently, a rotary-die equal-channel angular pressing (RD-ECAP) method [23,24] was proposed and confirmed to be a high-efficiency process for the application of RE-free Mg alloys [25,26,27,28,29]. As reported in Ref. [29], after 16 passes of the RD-ECAP treatment, an AZ91 alloy was obtained with a good tensile strength of 325 MPa and a balanced elongation to failure of 24%. However, the studies of application of this technology to Mg-RE alloy are seldom reported [30,31]. Moreover, the wear behavior of ECAP-processed Mg alloys is also a popular topic [32,33,34,35,36,37,38] worth investigating. 

In the present work, a Mg-10Gd-3Y (wt%) alloy (GW103) was processed by the RD-ECAP technique with a different number of passes to obtain a high-performance alloy with strength and ductility synergy. In the following sections, the microstructure and texture evolution based on different dynamic recrystallization mechanisms are discussed in detail; the friction behavior and the underlying mechanisms are also investigated. 

## 2. Materials and Methods

An as-cast Mg alloy with a nominal composition of Mg-10Gd-3Y (wt%) was produced from high-purity Mg (>99.7 wt%), Mg-30Gd (wt%) and Mg-30Y (wt%) master alloys. The casting process was carried out in an electric resistance furnace at about 760 °C, under a mixed gas atmosphere of SF_6_ and CO_2_. The chemical composition is illustrated in Table 1, which was measured using an inductive coupled plasma spectrometer analyzer.

The ingots were homogenized at 530 °C for 10 h, then quenched in warm water (about 60 °C) and prepared with a dimension of 20 mm × 20 mm × 45 mm for the rotary-die equal-channel angular pressing (RD-ECAP) process (same as the method in [27]). Before RD-ECAP, the bulk samples were pre-heated at 450 °C for 30 min. Subsequently, the RD-ECAP process was carried out at 450 °C followed by water quenching and the samples were processed by 1 pass (1P sample), 4 passes (4P sample) and 12 passes (12P sample), respectively. The channel angle Φ is 90° while the corner angle Ψ is 0°. The von Mises equivalent strains were calculated using Equation (1) and reported in Table 2.

(1) $$v-M\;\mathrm{strain}\;\mathrm{for}\;N\;\mathrm{passes}\;\mathrm{RD}-\mathrm{ECAP}:\;\;\bar{\varepsilon }=N\left[\frac{2\cot\left(\frac{\Phi }{2}+\frac{\Psi }{2}\right)+\Psi \mathrm{cosec}\left(\frac{\Phi }{2}+\frac{\Psi }{2}\right)}{\sqrt{3}}\right]$$

**Figure 1:**
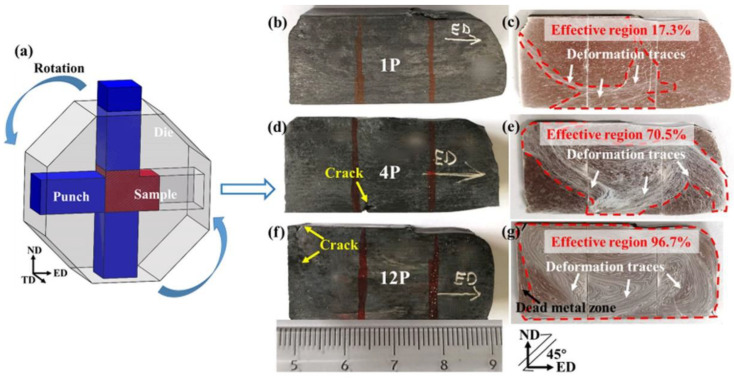
(**a**) Schematic of the RD-ECAP die (**b**,**d**,**f**), the macrographs of multi-pass RD-ECAP-processed sample, and (**c**,**e**,**g**) deformation traces revealed by metallographic corrosion, where the severe deformed zones were surrounded by red dotted lines.

The microstructures of the samples were observed using an optical microscope (OM, Axio Vert.A1), scanning electron microscope (SEM, FEI Quanta), and electron backscattered diffraction (EBSD) on the extrusion direction–normal direction (ED-ND) plane. The phase composition of all samples was measured via X-ray diffraction (XRD, X’Pert) with a scanning speed of 2.5° per minutes in range of 15°–80°. The samples for microstructural observation by OM and SEM were etched in a solution of 8% nitric acid and 92% ethanol after mechanical polishing. The macro textures of deformed samples were also measured via XRD. EBSD and macro texture results were analyzed using the software ATEX [39]. In order to study the mechanical properties in different process states, dog-bone-shaped tensile samples were prepared in the central part of the deformed samples along ED to ensure that the effective deformation sections (2 mm × 3 mm × 5 mm) of tensile samples were located in the effective deformed area (shown in Figure 1). The tensile tests were carried out on an electronic universal testing machine (AGS-X10KN) with a strain rate of 10^−3^ s^−1^. For each sample, the tensile test was performed in triplicate to ensure the accuracy of the mechanical properties. Friction tests were performed by a ball-on-disc reciprocating friction-and-wear testing machine (GF-Ι) for friction behavior investigation. Samples were mechanically ground to the mirror surface with a dimension of 2 mm × 10 mm × 13 mm, where the Al_2_O_3_ balls were applied as the counter-face material with a loading force of 10 N. Moreover, all the tests were carried out at room temperature with a velocity of 10 mm/s and a wear distance of 2 mm. Once the friction tests were completed, the testing surface was immediately cleaned with ethanol.

## 3. Results and Discussion

### 3.1. Microstructure Evolution

Figure 1 shows a schematic of the RD-ECAP process, macrographs of RD-ECAP-processed samples, and the related surface treated by metallographic corrosion. It can be observed that the 1P sample shows a significant change in appearance Figure 1b), while the 4P and 12P samples show a more regular rectangle shape (Figure 1d,f). Simultaneously, an obvious crack appears on the edge of the 4P sample, and a crack appears at the end of the 12P sample. After mechanical polishing, the surface of each sample shows deformation traces through metallographic corrosion, shown in Figure 1c,e,g. The deformation traces of 1P are consistent with typical flow patterns of a single pass of ECAP deformation as reported in [40,41], in which the effective region occupied around 17.3% of the sample. Based on the characteristics of the RD-ECAP process, the sample will be rotated after each pass [26,27]; consequently, the flow pattern is different from that of the typical routes A, Ba, Bc, and C [42]. As shown in Figure 1e, the deformation traces tilted toward multiple directions and formed a cycle around the center of the surface. The densest deformation traces around the position of the crack demonstrate that the formation of the crack is due to the dramatic plastic deformation in this area. More cycled traces can be found in the 12P sample (Figure 1g), where about 96.7% of the area was severely deformed. However, a dead metal zone [41] formed at the end of the 12P sample and resulted in the formation of cracking. 

**Figure 2:**
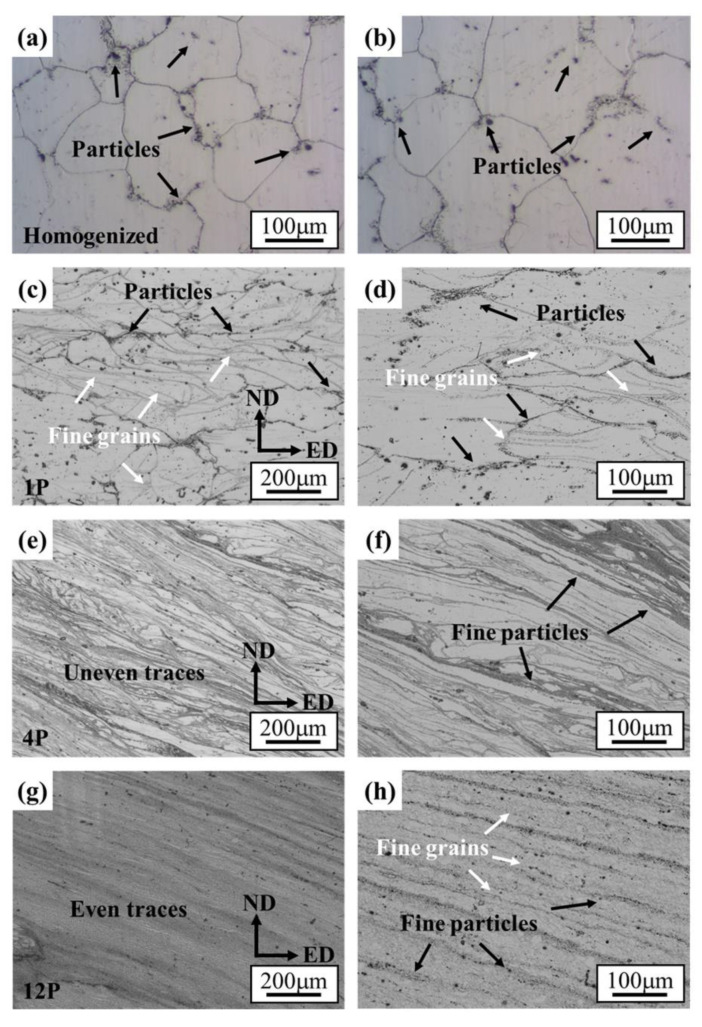
Optical micrographs of showing the microstructure evolution of (**a**,**b**) homogenized, (**c**,**d**) 1P, (**e**,**f**) 4P and (**g**,**h**) 12P samples.

**Figure 3:**
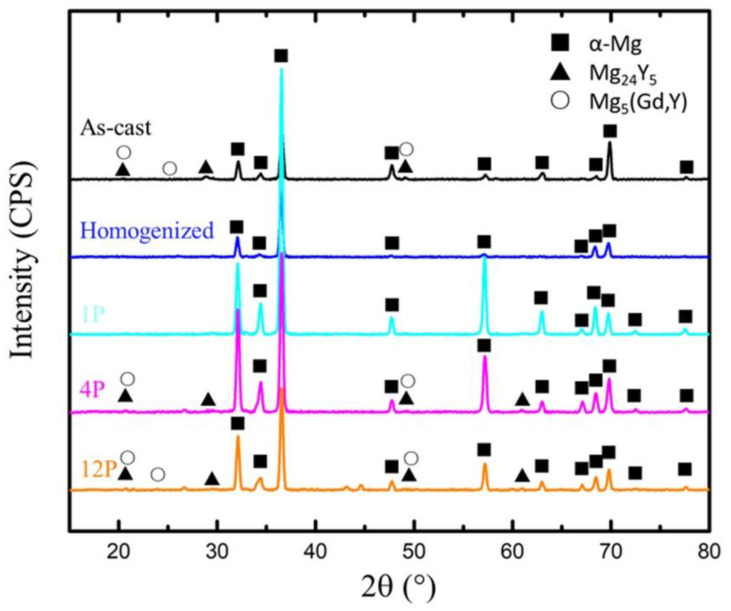
Phase composition of GW103 samples in different processing conditions.

Figure 2 presents the OM of the microstructures of the homogenized and RD-ECAP-processed samples. As shown in Figure 2a,b, the sample was homogenously treated at 530 °C for 10 h, and a microstructure with large equiaxed grains was obtained, the average grain size measured about 140 μm. Some insoluble particles can be observed, most of which are distributed along the grain boundaries (GB); only a few exist on the matrix. Similar results were reported in Ref [43,44,45]; with the addition of RE elements as Gd or Y, the segregation of particles will exhibit a tendency towards grain boundaries due to the large atomic size misfit with Mg and high bulk solubility. After a single pass of RD-ECAP, the grains were significantly elongated and oriented towards the ED due to the shear deformation at the junction of the channel (Figure 2c,d). Fine recrystallized grains appear at grain interiors or at some GB, which are generally clustered as belts. The particle distribution of the 1P sample is similar to the homogenized one, however, more particles can be observed near the GB. With the deformation increased to 4 passes, the microstructures were completely different. Figure 2e,f show that the microstructure of 4P sample was a combination of areas of elongated grains and uneven deformation traces. This indicates that the observed deformation traces in Figure 1 are mainly related to those refined particles induced by simple shear deformation during the RD-ECAP process. The recrystallized fine grains are difficult to observe by OM. For the 12P sample, the distribution of fine particles is dispersed and the deformation traces/flow lines become uniform (Figure 2g,h). The results show that a much finer microstructure with small grains and particles is aligned with the deformation flow lines, which presents evidence that the large, imposed shear strain accelerates fragmentation of the sample. Figure 3 shows the XRD-measured phase composition of samples in different conditions, and the Mg-RE particles were rarely detected in the homogenized sample which were more frequently found in the as-cast and RD-ECAP-processed samples.

**Figure 4:**
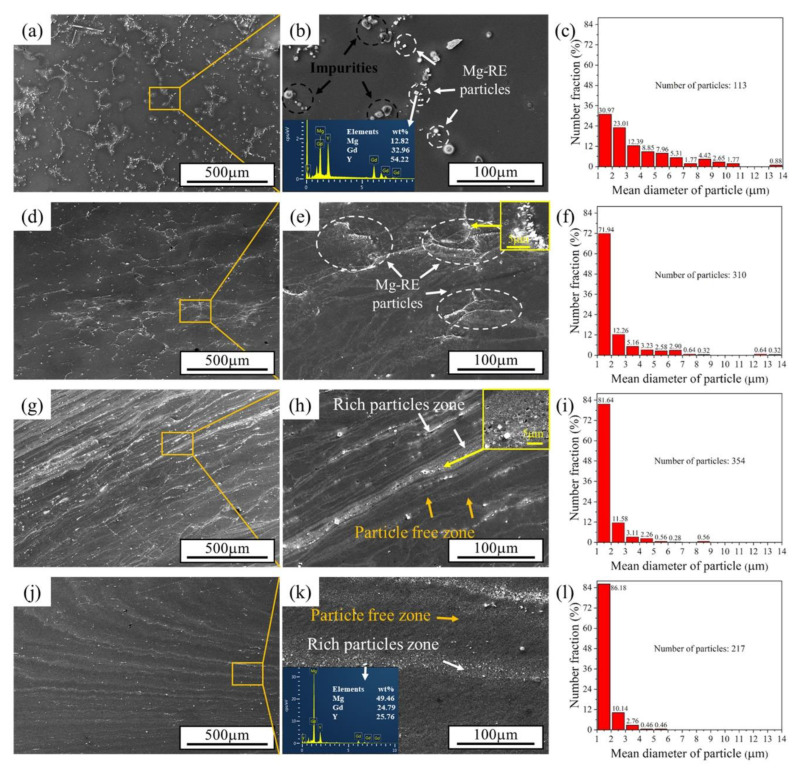
SEM morphologies in secondary electron signal (SE) of (**a**,**b**) homogenized, (**d**,**e**) 1P, (**g**,**h**) 4P, and (**j**,**k**) 12P samples; (**c**,**f**,**i**,**l**) the mean diameter distribution of particles (d > 1 μm) in maps (**b**,**e**,**h**,**k**).

SEM images are presented in Figure 4. SEM was performed to investigate the evolution of the spatial distribution and size of the particles during the RD-ECAP process. The mean diameter of coarse particles (diameter > 1 μm) was obtained by “image pro plus” software, and the size distributions are illustrated in Figure 4c,f,I,l. In the homogenized sample, shown in Figure 4a,b, the particles can be distinguished into two main types: the well-known cuboid-shaped Mg-RE particles [46] (in white circles) and impurities (in black circles), which are close to a spherical shape. The mean diameter of particles is larger as compared to the deformed samples; some particles even exceed 13 μm. As can be seen in Figure 4b,k, the composition of two particles is analyzed by energy-dispersive spectroscopy (EDS).

Figure 4d,e reveal that massive Mg-RE particles appear at the elongated grain boundaries of the 1P sample which were induced by dynamic precipitation. The impurities disappeared sharply and the grain size exhibited no obvious reduction after the first pass. The particles in the range of the mean diameter of 1 μm to 2 μm significantly increased from 30.97% to 71.94%, demonstrating that the ECAP process promotes the particle refinement. After 4 passes, the precipitate distribution and direction of the particle free zone are unfiled. Several coarse Mg-RE particles were located in the rich particles zone (Figure 4h); however, the mean diameter of all the particles decreased to less than 9 μm (Figure 4i). In addition, the local enlarged image of 4P sample in Figure 4h shows that the dynamic recrystallization (DRX) occurs in the rich particle zone, which is commonly observed during hot ECAP deformation for precipitates contained Mg alloy [29,47]. After 12 passes, the particles are further refined and show a more dispersed distribution around the rich particle zone. As can be seen in Figure 4l, almost all the particles have a small mean diameter of less than 2 μm. A similar particle evolution is reported by Lu et al. [48], where the Mg-Zn-Gd-Zr alloy was processed by multi-pass ECAP. 

**Figure 5:**
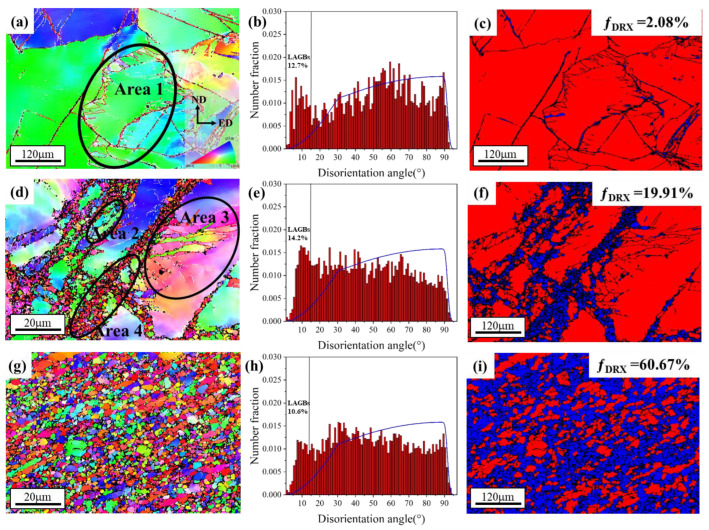
EBSD IPF maps with ED projected for (**a**) 1P, (**d**) 4P and (**g**) 12P samples; (**b**,**e**,**h**) show the disorientation angle distribution. Recrystallized area fractions of deformed samples are shown in (**c**,**f**,**i**), where the DRX grains are in blue and the deformed grains are in red.

EBSD measurements were performed to investigate the further microstructural evolution during the RD-ECAP process. Figure 5 presents the EBSD inverse pole Figures (IPF) maps, disorientation angle distributions and recrystallized area fraction maps of 1P, 4P and 12P samples. The DRX grains are those displaying an average internal disorientation of less than 2° and where 3° is never exceeded in the grain. The internal disorientation is calculated by taking the average of the disorientation between the average orientation of the grain and the orientation of each pixels constituting the grains. The grain tolerance angle was set to 5°.

The low-angle grain boundaries (LAGB; 2° ≤ θ ≤ 15°) and high-angle grain boundaries (HAGB, θ > 15°) are defined, which were highlighted by thick white and red lines, respectively. As shown in Figure 5a, coarse grains induced by homogenized treatment were not refined and a large number of dynamic recrystallized (DRX) fine grains formed along the GBs and inner of coarse grains. Some sub-grain boundaries (sub-GB) with a disorientation angle exceeding 2° can be clearly seen in the coarse grains which will promote the DRX process by absorbing the dislocation during the deformation [9,49]. Due to the presence of a high density of dislocations, internal grain disorientation appears to result in the variation in color of the IPF maps. The same phenomenon is also shown in 4P (Figure 5d) and 12P (Figure 5g) samples in the deformed grains. After 4 passes, the sample shows a typical bimodal microstructure [9,50,51], where coarse deformed grains are surrounded by massive fine DRX grains. The area fraction of recrystallization grains reached 19.91% as shown in Figure 5f, which is increased by 17.83% compared to the 1P sample (Figure 5c). It is worth mentioning that the distributions of recrystallized fine grains in both 1P and 4P are highly unified with the Mg-RE particle distribution (Figure 4), where the fine grains prefer to distribute along the grain boundaries of deformed grains or form the fine grain bands that split the matrix. Figure 5g,i present the microstructure of the 12P sample; the coarse matrix was significantly refined and more DRX grains formed, where the average grain size of this sample was ~1.3 μm. However, the dynamic recrystallization is still not completed and the area fraction of DRX is 60.67%. Moreover, Figure 5h presents a more random distribution of the disorientation for 12P with the lowest LAGB fraction compared to 1P and 4P (Figure 5b,e).

**Figure 6:**
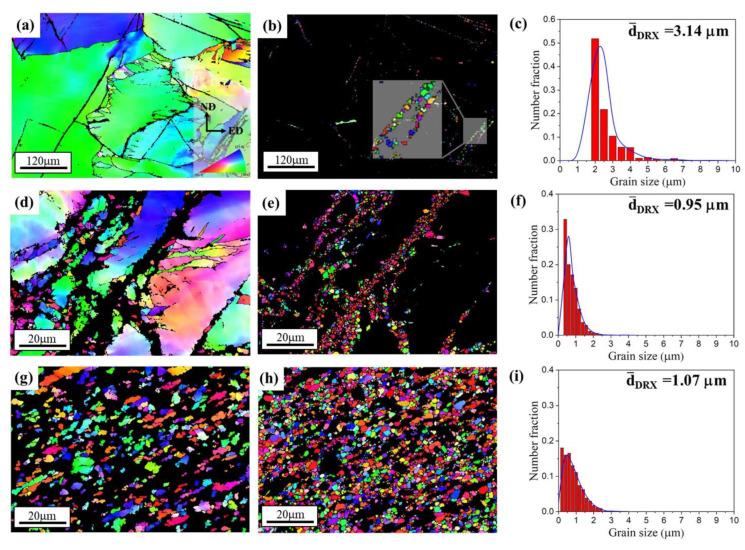
Separated EBSD maps of (**a**,**d**,**g**) deformed grains and (**b**,**e**,**h**) DRX grains. The grain size distribution of DRX grains is presented in (**c**,**f**,**i**) by the number fraction.

To better understand the grain refinement mechanism, the EBSD IPF maps of deformed grains and DRX grains for 1P, 4P and 12P samples are spilt into two parts: DRX and deformed grains (Figure 6). The results reveal that the orientation of deformed grains is gradually changed with the increase in imposed shear strain; as shown in Figure 6d,g, a growing number of deformed grains shows a different orientation with the coarse matrix in Figure 6a. For the DRX grains in Figure 6e,h, the orientations seems to be random. Concerning the grain size, the grain size distribution of DRX grains is shown in Figure 6c,f,i. The average grain size of DRX grains decreases from ~3 μm to ~1 μm when the RD-ECAP is between 1P and 4P. As the RD-ECAP processes were carried out at relative high processing temperature of 450 °C, the DRX occurs along with the grain growth. Figure 6f,i show that the average grain size of DRX grains in 12P sample is slightly higher than that in the 4P sample, and the number fraction of fine DRX grain (<1 μm) is lower in the 12P sample. The new orientation formation of deformed or DRX grains are strongly related to the DRX behavior during the RD-ECAP process, and it will have an obvious impact on the texture. Therefore, the detailed analysis of the DRX mechanism and its effect on the related texture will be proposed with the texture evolution analysis in Section 3.2, based on the selected area 1 to 4 in Figure 5.

### 3.2. Texture Evolution

**Figure 7:**
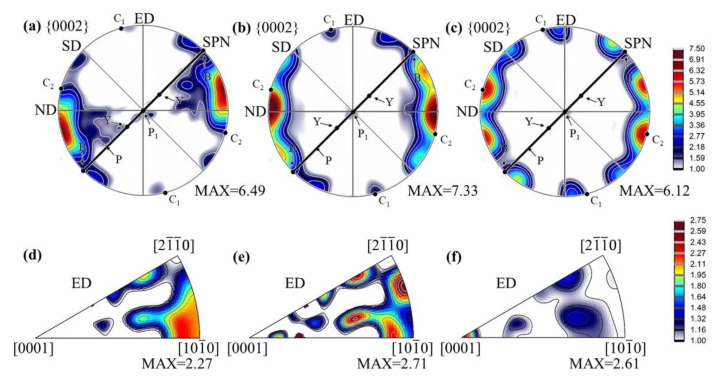
Texture evolution in {0002} pole Figures and IPF based on XRD: (**a**,**d**) 1P, (**b**,**e**) 4P and (**c**,**f**) 12P.

The macrotexture evolution of RD-ECAP processed alloys was measured by using XRD on the ED-TD plane; the results are presented in Figure 7 in the form of {0002} pole Figures and IPF of ED. Figure 7a shows the texture of 1P sample; the ideal orientations for 90° angle die ECAP-processed Mg alloy with the shear direction (SD) at 45° are labeled on the pole Figure and recalled in Table 3, which are called as B, P, Y C_1_ and C_2_ [52,53,54]. The texture components B and P are considered to be the most significant [52] in magnesium under simple shear. The results reveal that the texture evolution of 1P sample basically follows the ideal state; the grains orientations mainly rotate anti-clockwise around TD to accumulate in the vicinity of the B fiber. The maximum intensity is located between the B and C_2_ fiber texture about 23° away from the shear plane normal (SPN). The deviation is mainly induced by the RE elements and/or inhomogeneous deformation. A weak C_1_ component can be found in the 1P sample, which will be gradually increased and trend to be parallel to ED with the increasing imposed strain, as shown in Figure 7b,c. Nevertheless, more components are formed as shown in the pole Figures of 4P and 12P samples, where the texture do not fit the ideal textures. A similar texture evolution was reported in Ref. [25] for multi-pass RD-ECAP-processed AZ91 alloy; the sample rotation between RD-ECAP passes result in the formation of the bi-texture components constituted by basal and prismatic texture components. Regarding the texture maximum, it is increased from 6.49 to 7.33 between 1 pass and 4 passes, and subsequently decreased to 6.12 after 12 passes.

The IPF in Figure 7d presents a strong texture component of <10$\bar{1}$0 >//ED for 1P sample. The texture components of 1P sample are mainly provided by the deformed coarse grains as shown in Figure 5 and Figure 6, the surface fraction of DRX grains only occupied 2.08% which has limited influence on the texture. The 4P sample presents a random texture distribution as shown by IPF in Figure 7e. For the 12P sample, as seen in Figure 7f, the texture was weakened and a new texture component with c-axis parallel to the ED formed. These “abnormal texture” components have been reported in Refs. [55,56,57], it has been defined as “C-texture”, which was usually observed in extruded Mg alloy with a high extrusion ratio and/or in the extruded Mg alloy with RE contained.

**Figure 8:**
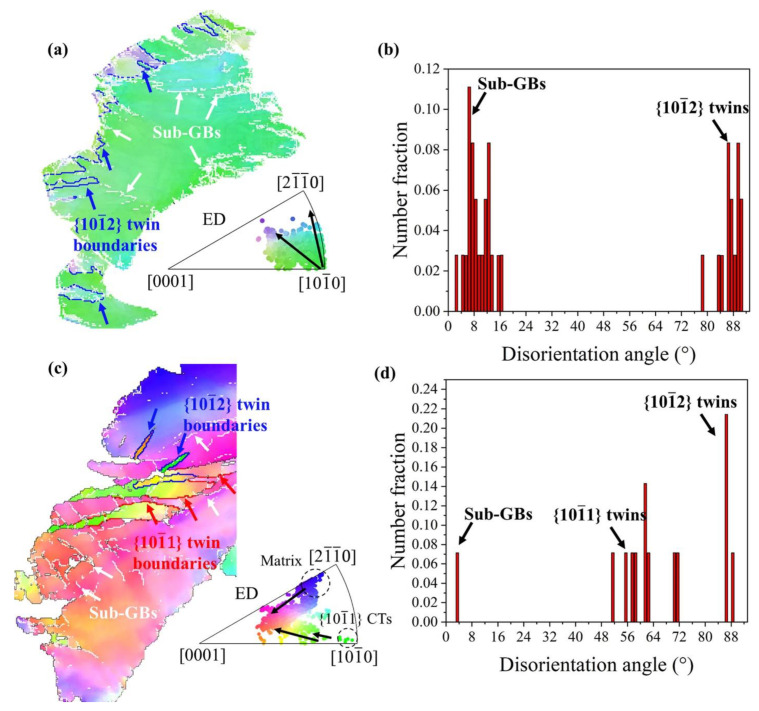
Orientation evolution of deformed matrix: (**a**) deformed grains of area 1, (**c**) deformed grains of area 3; (**b**,**d**) related disorientation angle distributions.

**Figure 9:**
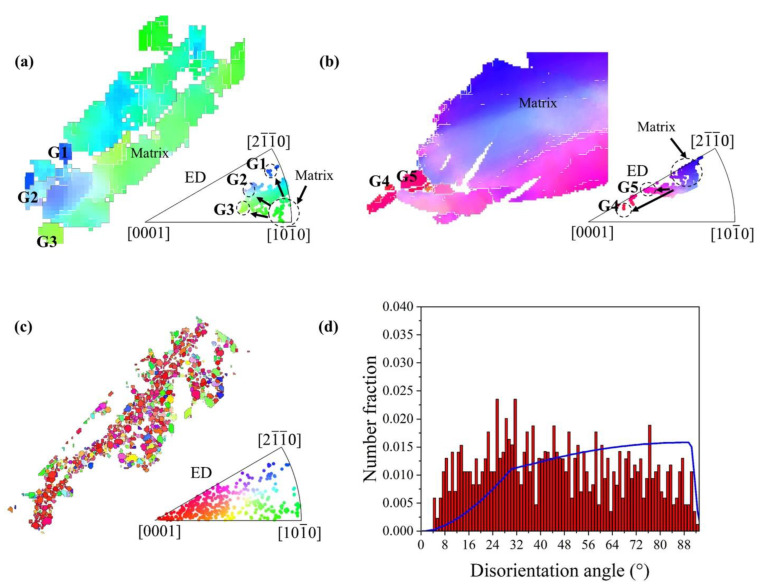
Dynamic recrystallization mechanism analysis based on the selected area 1–4: (**a**,**b**) CDRX, (**c**) fine DRX grains and (**d**) disorientation angle distribution of fine DRX grains in (**c**).

In order to further investigate the texture evolution during the RD-ECAP process, several representative areas were selected from Figure 5 and presented in Figure 8 and Figure 9. As the texture transformation is strongly related to the DRX during the hot deformation, the typical twin induced dynamic recrystallization (TDRX), continuous dynamic recrystallization (CDRX) and discontinuous dynamic recrystallization (DDRX) mechanism are systemically analyzed based on the EBSD results of 1P and 4P samples. Figure 8a shows a deformed zone of 1P sample, where massive sub-GB and {$10\bar{1}2$} extension twins (ET) can be observed. It is obvious that the area surrounded by sub-GB and/or twin boundaries have different color gradients with the matrix, which is the orientation gradient induced by the related deformation, where sub-GB in the interior of the matrix represents the process of CDRX [58]. Simultaneously, TDRX occurs with the high dislocation activity within or at the {10$\bar{1}$2} twin boundaries; the accumulated dislocations near the boundaries provide the nucleation sites for new DRX grains [59]. The direct projection IPF plot shows the same results in Figure 7d, which confirms the formation of {10$\bar{1}$2} ETs and sub-GB induced by reorientation of the local matrix from <10$\bar{1}$0> to <2$\bar{1}\bar{1}$0>. At the early state of 1P, DDRX is considered as the main DRX mechanism, where the serrated GB of matrix provides the nucleation site for DDRX [58,59] and the dense Mg-RE particles along the GB promote the DDRX by the particle stimulate mechanism (PSN) [60,61]. As can be observed in Figure 6b, the fine grains induced by DDRX have no preferred orientations [9]. The related disorientation angle distribution of area 1 is shown in Figure 8b. With the imposed strain increased to 4.6 of 4P, more disorientation appears in the deformed matrix seen in Figure 5d. Figure 8c,d reveal that both {10$\bar{1}$2} ET and {10$\bar{1}$1} compression twins (CT) can be activated in the 4P sample. As shown by Jiang et al. [58], which carefully studied the shear effective zone of extruded AZ31 alloy, {10$\bar{1}$1} CT and <10$\bar{1}$0> fiber texture appeared after the trigger of {10$\bar{1}$2} ET due to its higher critical resolve shear stress (CRSS) and TDRX can be easily induced by {10$\bar{1}$1} ET and {10$\bar{1}$1}–{10$\bar{1}$2} double twins. In our work, the formation of a large number of sub-GB in the {10$\bar{1}$1} CT confirms the reported results. Under this condition, the matrix shows a reorientation tendency from <2$\bar{1}\bar{1}$0> fiber to <0001> basal fiber, and the orientation of {10$\bar{1}$1} CT shows a reorientation tendency of <10$\bar{1}$0> fiber towards <0001> basal fiber. Finally, more texture components formed with weak intensity and the strong initial <10$\bar{1}$0> basal texture weakened.

Figure 9a,b present the typical CDRX [9,58,59] process in the observed EBSD results of 4P sample. As shown in Figure 9a the DRX grains of G1–G3 have several reorientation directions marked with black narrows which are similar with the matrix reorientation in Figure 8a. The orientation of G1 is near the <2$\bar{1}\bar{1}$0>//ED fiber, which reveals that the <2$\bar{1}\bar{1}$0>//ED fiber texture can be provided by CDRX. Similarly, Figure 9b shows the DRX grains G4–G5 trend to form the <0001>//ED fiber when the initial orientation of the matrix is <2$\bar{1}\bar{1}$0>//ED. In the rich Mg-RE particle zone (Figure 4h), it is evident that the DRX fine grains were not only formed by DDRX; the DRX fine grains induced by CDRX possibly appear in this area. Therefore, it is hard to define the DRX mechanism of those fine grains in area 4 (Figure 9c). However, it can be clearly seen that the majority of grains are oriented in <0001>//ED. For the final texture obtained in 12P sample (Figure 7f), it can be concluded that the formation of C-texture is related to both CDRX and DDRX; the absence of <10$\bar{1}$0> basal fiber can be attributed to the reorientation of the matrix and CDRX grains. Both reorientations of matrix and twins induced by RD-ECAP have the texture randomization effect on the processed samples, which is also the main reason in the weakening of basal texture of those samples as shown in Figure 7.

### 3.3. Mechanical Properties

**Figure 10:**
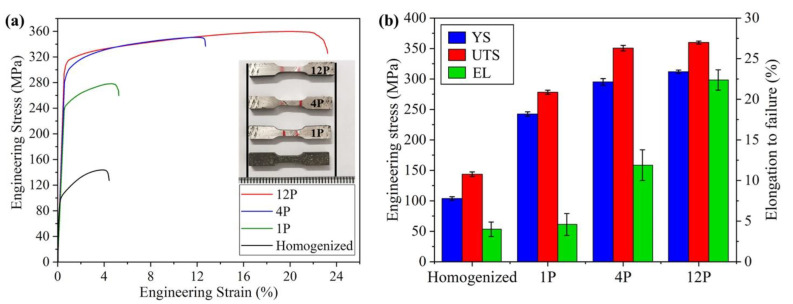
(**a**) Engineering stress–strain curves of GW103 samples after different processing conditions; (**b**) the corresponding mechanical properties: yield stress (YS), ultimate tensile strength (UTS) and elongation to failure (EL).

Figure 10 displays the tensile stress–strain curves of the GW103 alloy after different processing conditions. The results reveal that RD-ECAP is an effective process to simultaneously achieve the enhancement of both the strength and ductility of GW103 alloy. After the 1P process, the yield stress (YS) was significantly increasing from 104 MPa to 243 MPa with the ultimate tensile strength (UTS) increased from144 MPa to 278 MPa. However, the elongation to failure (EL) has barely increased, which is due to the limited DRX and clustered coarse Mg-RE particles along GBs as shown in Figure 2 and Figure 4. When the alloy was treated by 4P, the particle size was refined (Figure 4); and a bimodal microstructure formed with more DRX fine grains (Figure 5), as a consequence both the strength and ductility were improved, where the YS, UTS and EL are 295 MPa, 351 MPa and 12%, respectively. For 12P sample, the YS and UTS showed no obvious improvement, which were 312 MPa and 360 MPa. However, the highest EL of 22% was obtained.

In general, the grain refinement induced grain boundary strengthening, second-phase precipitate strengthening and texture strengthening are considered to affect the mechanical properties of hot-deformed Mg alloys [25,26,55,62]. In this study, the high imposed strain in 12P processed sample under high temperature (450 °C) condition led to a radical change in both the microstructure and texture. The average grain size of GW103 alloy was dramatically refined from ~140 μm to ~1.3 μm after being processed by 12P. Based on the well-known Hall–Petch relationship, the strength of the alloy could be improved greatly. Simultaneously, the diffusely distributed fine Mg-RE particles induced by shear strain at grain boundaries and triple junction could further enhance the strength with related to the well-known precipitation strengthening based on Orowan theory. Additionally, compared with the typical ECAP texture, the new bi-texture with prismatic slip induced texture gradually formed during RD-ECAP process where the texture of 12P sample shows more components with a relatively lower intensity (Figure 7c). Therefore, the high ductility performance of 12P sample is attributed to the drastic grain refinement and the reduction or randomization of the texture induced by DRX. The combination of C-texture formation and weakening of basal texture benefit the ductility of GW103 alloy. Especially, tensile twins are reported to be observed in the grains with C-texture [55,57,63], which could also improve the ductility. To compare with several RD-ECAP-processed AZ91 alloy reported in Refs. [25,26,27,29] and a Mg-Y-Er-Zn alloy reported in [30], the 12P sample in the present study shows the best combination of high strength and high ductility by comparing to the sample processed by the same shear strain level. The main reasons are that: (1) significant grain refinement induced by DRX plays a role in grain boundary strengthening; (2) fine Mg-RE particles induced precipitate strengthening; (3) the multiple texture components with lower intensities were achieved in the processed GW103 sample. This demonstrates that the RD-ECAP is an effective method in processing high-performance Mg-Gd-Y alloys.

### 3.4. Friction Behavior

**Figure 11:**
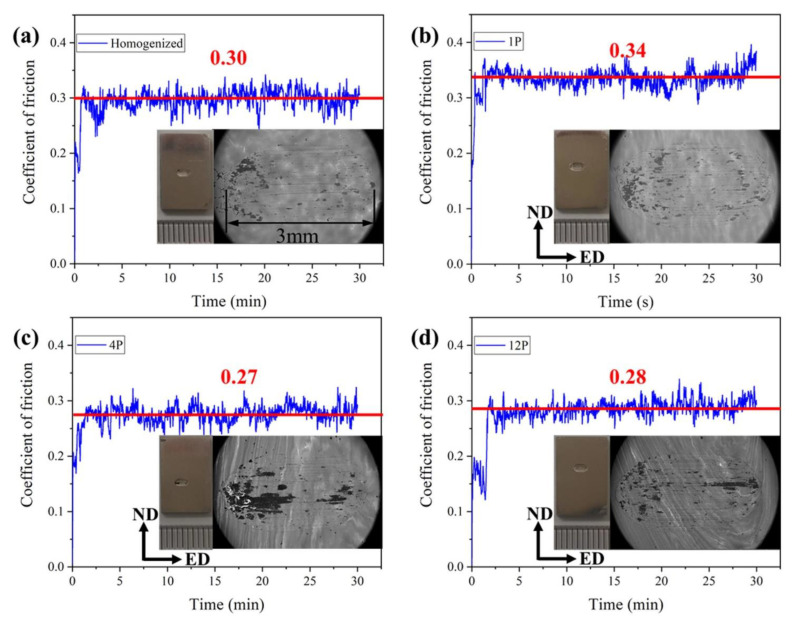
Coefficient of friction fluctuation with time for (**a**) Homogenized and (**b**) 1P; (**c**) 4P; (**d**) 12P processed samples with the corresponding macrographs and SEM images, where the SEM images are in backscattered electron signal (BSE).

The fluctuations of the coefficient of friction (COF) versus testing time are shown in Figure 11 for the samples with different processing states. The related macrographs and SEM morphologies in BSE of wear areas are also presented in the Figures. The friction testing was carried out at a 10 N loading, and the sliding direction is along the ED. For 1P, 4P and 12P samples, it can be observed that the testing zone is in the effective RD-ECAP deformed zone based on the precipitate distribution in and Figure 4 and Figure 11b–d. The results reveal that the average COF varies from processing conditions. When the sample was treated by the first pass, the highest COF of 0.34 was obtained. However, with the increasing imposed strain, the COF decreased to 0.27 and 0.28 for 4P and 12P samples, respectively. The COF of multi-pass RD-ECAP-processed sample is lower than the homogenized sample (0.3).

As reported in several studies [32,35,36,37,64], the COF of the alloy is normally affected by the coordination of multiple factors including the grain size, precipitates, loading force and sliding speed, etc. Shanthi et al. [64] studied the effect of the grain size and sliding speed of the AZ91 alloy; the load was the same as in this study: 10 N. They found that the grain size has no obvious effect on the wear performance. Therefore, the grain size is not considered as the main factor to influence the COF in this work.

**Figure 12:**
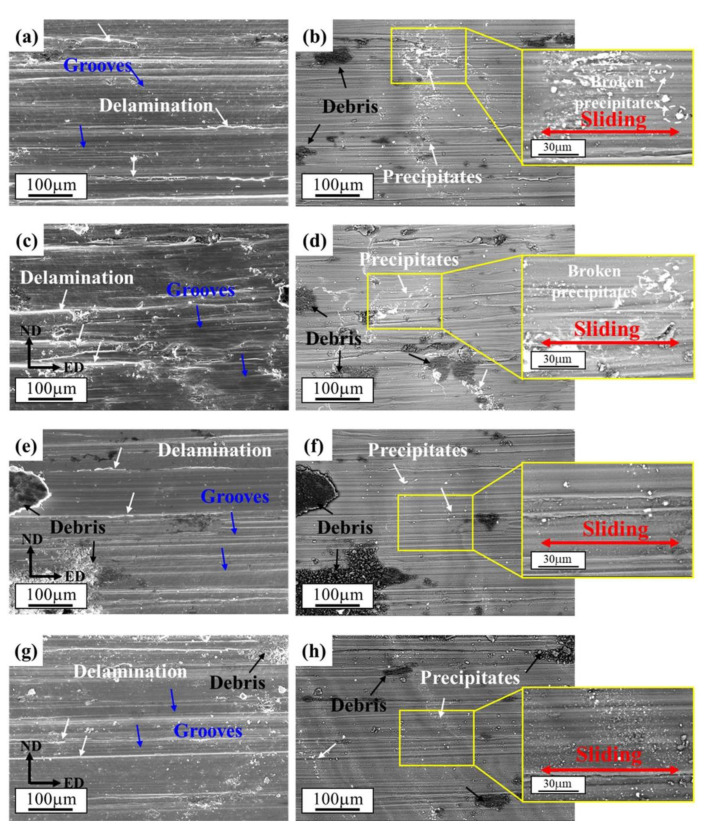
SEM morphologies of worn surfaces after friction testing in different signals: (**a**,**b**) homogenized, (**c**,**d**) 1P, (**e**,**f**) 4P and (**g**,**h**) 12P samples. Images of (**a**,**c**,**e**,**g**) are in SE and (**b**,**d**,**f**,**h**) are in BSE.

Figure 12 presents the worn surfaces of the different processed alloys. Typical characteristics of delamination, grooves and debris [33,35] can be observed in all the samples. However, the observed rough surface of 1P sample (Figure 12c) seems to have dramatically deformed. A similar phenomenon was reported in [32] for ECAP-processed Mg-Sn alloys; due to the high dislocation density and stress field in ECAP process, the oxidation rate will quickly occur during the wear test. The mechanism will change from abrasive wear to oxidative wear in the ECAP-processed samples with high dislocation density and stress field. However, the RD-ECAP process was performed at a high temperature of 450 °C; for 4P and 12P samples, there exist rare residuary dislocation and stress fields. Therefore, the wear mechanisms of 4P and 12P samples are similar with the homogenized sample. As the precipitates are another important influencing factor during the friction testing [65]; SEM images in BSE are shown in Figure 12b,d,f,h. The precipitates distributions of the homogenized sample and 1P sample are observably changed compared to that in Figure 4. The clustered precipitates were broken and scattered during the friction testing for the two samples, as shown Figure 12b,d; the precipitates are no longer distributed along the grain boundaries which are dispersed with a new distribution tendency along the sliding direction. For 4P and 12P samples, the appearance and distribution of precipitates was maintained after being processed. The results demonstrated that the precipitates could hinder the friction during the test. According to the particle size and distribution in Figure 4 and Figure 12 and the curves of COF in Figure 11, it can be concluded that the refinement and dispersion of precipitates during the shear deformation could also benefit the reduction in the COF.

## 4. Conclusions

In this study, a homogenized GW103 alloy was deformed by 1 pass, 4 passes, and 12 passes of the RD-ECAP process at 450 °C. The microstructure, texture evolution, mechanical properties and friction behavior were systemically investigated. The following main conclusions can be drawn:

(1) With the increase in imposed strain during the RD-ECAP process, the grain size of the homogenized GW103 alloy was refined from ~140 μm to ~1.3 μm after 12 passes. A dispersed distribution of fine particles (~1 μm) was obtained.

(2) Dynamic recrystallization (DRX) is the main grain refinement mechanism during the 450 °C RD-ECAP process, where continuous DRX, discontinuous DRX and twining-induced DRX were commonly observed, which can also induce multiple texture components for the randomization of ECAP textures.

(3) Both high strength and high ductility were achieved simultaneously via 12 passes of RD-ECAP, where the YS, UTS and EL reached 312 MPa, 360 MPa, and 22%, respectively. Grain refinement, precipitate evolution, and texture weakening contributed to those high performances.

(4) A friction coefficient under 10 N loading can be enhanced by a single pass of RD-ECAP; however, it will be reduced by the following multi-pass hot deformation at 450 °C.

## Figures and Tables

**Table 1:** Chemical composition of as-cast GW103 sample.

Composition	Gd	Y	Al	Zn	Fe	Mg
Content/wt%	10.62	3.21	0.005	0.01	0.003	Bal.

**Table 2:** Testing information and the related strain.

Sample	Processing Temperature (°C)	RD-ECAP Channel Angle Φ (°)	RD-ECAP Corner Angle Ψ (°)	von Mises Strain
1P	450			1.15
4P		90	0	4.60
12P				13.80

**Table 3:** Ideal orientations for Mg alloy during ECAP with die angle of 90° [52]. Reprinted with permission from Ref. [52]. 2008; Elsevier.

Fiber	*φ*_1_ (°)	Φ (°)	*φ*_2_ (°)
B	45	90	0–60
P	45	0–90	30
Y	45	30	0–60
C_1_	105	90	0–60
C_2_	105	90	0–60

## Data Availability

Not applicable.
